# Effects of Oxygen Tension for Membrane Lipidome Remodeling of Cockayne Syndrome Cell Models

**DOI:** 10.3390/cells11081286

**Published:** 2022-04-10

**Authors:** Carla Ferreri, Anna Sansone, Marios G. Krokidis, Annalisa Masi, Barbara Pascucci, Mariarosaria D’Errico, Chryssostomos Chatgilialoglu

**Affiliations:** 1 Istituto per la Sintesi Organica e la Fotoreattività, Consiglio Nazionale delle Ricerche, Via P. Gobetti 101, 40129 Bologna, Italy; carla.ferreri@isof.cnr.it (C.F.); anna.sansone@isof.cnr.it (A.S.); annalisa.masi@ic.cnr.it (A.M.); 2 Institute of Nanoscience and Nanotechnology, N.C.S.R. “Demokritos”, Agia Paraskevi Attikis, Athens 15310, Greece; m.krokidis@inn.demokritos.gr; 3 Institute of Crystallography, Consiglio Nazionale delle Ricerche, Monterotondo Stazione, 00015 Rome, Italy; barbara.pascucci@ic.cnr.it; 4 Department of Environment and Health, Istituto Superiore di Sanità, Viale Regina Elena 299, 00161 Rome, Italy; mariarosaria.derrico@iss.it; 5 Center for Advanced Technologies, Adam Mickiewicz University, 61-614 Poznań, Poland

**Keywords:** CSA, CSB, oxygen concentration, membrane fatty acids, monounsaturated fatty acids, polyunsaturated fatty acids, membrane homeostasis indexes

## Abstract

Oxygen is important for lipid metabolism, being involved in both enzymatic transformations and oxidative reactivity, and is particularly influent when genetic diseases impair the repair machinery of the cells, such as described for Cockayne syndrome (CS). We used two cellular models of transformed fibroblasts defective for CSA and CSB genes and their normal counterparts, grown for 24 h under various oxygen tensions (hyperoxic 21%, physioxic 5% and hypoxic 1%) to examine the fatty acid-based membrane remodeling by GC analysis of fatty acid methyl esters derived from membrane phospholipids. Overall, we first distinguished differences due to oxygen tensions: (a) hyperoxia induced a general boost of desaturase enzymatic activity in both normal and defective CSA and CSB cell lines, increasing monounsaturated fatty acids (MUFA), whereas polyunsaturated fatty acids (PUFA) did not undergo oxidative consumption; (b) hypoxia slowed down desaturase activities, mostly in CSA cell lines and defective CSB, causing saturated fatty acids (SFA) to increase, whereas PUFA levels diminished, suggesting their involvement in hypoxia-related signaling. CSB-deprived cells are the most sensitive to oxidation and CSA-deprived cells are the most sensitive to the radical-based formation of trans fatty acids (TFA). The results point to the need to finely differentiate biological targets connected to genetic impairments and, consequently, suggest the better definition of cell protection and treatments through accurate molecular profiling that includes membrane lipidomes.

## 1. Introduction

Phospholipids, which are composed of two hydrophobic fatty acid (FA) moieties and one hydrophilic head group, are major constituents of biological membranes. The chain length and the number of double bonds of the two FA moieties determine the biophysical properties of membranes that have a strong impact on biological processes [1]. Membrane fatty acid remodeling is a process known as Lands’ cycle [2], which occurs as an immediate response to changes of the cell culture environment, by the sequential intervention on the membrane phospholipids of phospholipase A2 (PLA2) and lysophospholipid acyltransferase (LPCAT) [1]. Several PLA2s and LPCATs have been identified and characterized since the late 1980s [1]. Membrane remodeling is activated by a large variety of conditions that some of us have experienced in cell cultures, e.g., nutrient concentrations such as glucose [3], fatty acids [4], and antioxidants [5]; and pharmacological interventions [6,7,8,9].

The oxygen level is an environmental condition that can cause membrane remodeling. This is particularly evident when the ischemia–reperfusion process occurs and causes an injury due to several factors connected with the return of oxygen flow in tissues and the impairment of the so-called antioxidant network [10]. It is worth mentioning that membrane remodeling can also occur as a pure “mechanical” process for an instantaneous readaptation of the surface and volume in response to perturbations, such as wound healing, breathing or any other conditions that cause cell shape variations, thus involving plasma membrane [11]. Overall, oxygen tension can trigger the fast adaptation response driven by such physical conditions and rearrangement of the lipid pool, caused by enzymatic, metabolic and chemical (oxidative–redox) responses that influence the availability of the fatty acid molecules for membrane phospholipid remodeling. In fact, in fatty acid biosynthesis, desaturase enzymes are key steps that require an oxygen molecule, so that the levels of unsaturated fatty acids (UFA) can also be influenced by oxygen tension [12]. The study of membrane lipidomics in cell culture experiments under various conditions provides information on the maintenance and turnover of the cell membrane, a specific and necessary compartment for physiological functioning [13].

**Figure 1:**
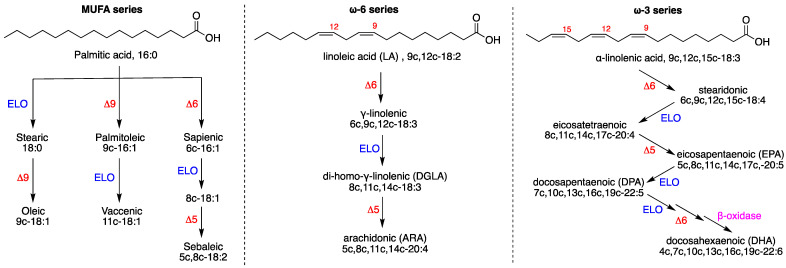
Biosynthesis of fatty acids (FA): (**left**) biosynthetic pathways of MUFA, starting from palmitic acid; (**center**) the omega-6 PUFA biosynthesis starting from linoleic acid; (**right**) the omega-3 PUFA biosynthesis starting from alpha-linolenic acid. Enzymes: ELO elongase; Δ5-, Δ6- and Δ9-desaturase; β-oxidase. Numerical abbreviations describing the position and geometry of the double bonds (e.g., 9c), as well as the notation of the carbon chain length and total number of unsaturation (e.g., C18:2); in parentheses are the acronyms used in this work (e.g., ARA for arachidonic acid).

Figure 1 shows the biosynthesis of monounsaturated fatty acids (MUFA) and polyunsaturated fatty acids (PUFA) together with examples of their structures. Starting from the saturated fatty acid (SFA) palmitic acid, which is obtained by the fatty acid synthase, the alternation of elongases, Δ9 or Δ6 desaturase enzymes, creates a variety of MUFA, thus mitigating the effect of SFA with the formation of double bonds that contribute to cell membrane fluidity [14,15]. The ω10 fatty acid pathway from sapienic acid (Figure 1, left side) is a recently examined route in cancer metabolism [16]. PUFA are essential for eukaryotic cells, and their biosynthetic pathways start from the dietary intakes of the omega-6 and omega-3 FA precursors, linoleic and alpha-linolenic acids, respectively.

In Figure 1, the FA nomenclature only shows the cis isomers of unsaturated molecules (i.e., the 9c in oleic acid 9c-18:1 means cis configuration in 9-position). It is worth noting that the geometrical isomers of natural FA can be formed (i.e., 9t-18:1) during oxidative stress, due to the cis–trans isomerization process mediated by radical species [6,13]. Our based membrane lipidomics approach provides the accurate determination of FA changes occurring in the pool and reflected in the remodeling of membrane phospholipid composition, including positional isomers (such as 6c-16:1 and 9c-16:1) as well as geometrical isomers (such as 6c-16:1 and 6t-16:1) that cannot be performed by techniques using mass detection, since the molecular masses of the geometrical and positional isomers are the same [6,13].

Indeed, oxygen tension is becoming an important variable to study in cell experiments, in order to evaluate the mechanisms of adaptation that allow survival, thus unveiling molecular mechanisms inspiring rescue and therapeutic strategies in diseases such as cancer [17,18], obesity [19,20] and diabetes [19,21]. On the other hand, oxidation processes by reactive oxygen species (ROS) challenge the cellular antioxidant network, and at lower concentrations of ROS, normal cellular signaling occurs; however, when concentrations are higher and time exposure becomes long, extensive damage to cellular macromolecular content (DNA, lipids and proteins) can happen [22]. For the chemical integrity of tissues such as brain, where neuronal membranes are found to be rich in PUFA, oxygen and the consequent oxidative reactivity are important, and various neurodegenerative diseases, such as Parkinson’s disease and Alzheimer’s disease, present alterations in the molecular content due to oxidative stress [23,24,25,26]. Oxidation processes can be even more harmful in genetic diseases such as Cockayne syndrome (CS), which involves nucleotide excision repair (NER), a process that removes a variety of DNA-blocking lesions [27].

CS is cancer-free and its clinical features are pre- or post-natal growth failure, leading to a characteristic appearance of so-called cachectic dwarfism, premature aging and progressive neurological dysfunction. CS proteins have a role in several biological processes and this could explain the pleiotropic and variable phenotype of CS patients. Over 90% of CS cases are due to mutations in either the Cockayne syndrome A (CSA) or Cockayne syndrome B (CSB) genes. CS cells are defective in the removal of lesions in the transcribed strand of actively transcribed genes (transcription-coupled NER sub-pathway, TCR) [27]. Because the total loss of NER is not necessarily followed by developmental impairment and premature aging, alternative concepts are also under investigation [28].

CS cells, in addition to being hypersensitive to UV light, are also hypersensitive to several types of oxidative DNA-damaging agents considering that CS proteins stimulate the activities of key base excision repair enzymes and/or affect their transcription. CS proteins are involved in the recognition, signaling and processing of single-strand breaks as well as double-strand breaks [29,30,31]. Moreover, CS proteins have a role in chromatin remodeling, RNAPII processing, nucleolin regulation and rDNA and rRNA transcription [32,33], as well as in protecting cells from senescence [34,35]. Finally, it was also proposed that the loss of proteostasis as well as mitochondrial dysfunction may play an important role in the etiology of CS [36,37,38,39]. From this scenario, it emerges how important it is to examine molecular unbalances as consequences of oxidative conditions, which can have a much more profound impact on the diseased cells compared to the healthy ones. This knowledge can lead to individuated biological targets and can help to plan specific protective and therapeutic strategies.

In a recent study, we investigated the role of oxygen on the formation of 5′,8-cyclopurines, which are DNA lesions exclusively caused by free radical reactivity and not to be confused with oxidative lesions such as 8-oxo purines. In cellular models of defective CSA- and CSB-transformed fibroblasts and their normal counterparts, grown under various oxygen tensions (hyperoxic 21%, physioxic 5% and hypoxic 1%), we observed that higher levels of these adducts are present under hypoxia in both CSA- and CSB-defective cells compared to normal cells [40]. Furthermore, we also provided for the first time a parallel analysis on both genome and membrane molecular transformations in human embryonic epithelial cells silenced for XPA using hyperoxic conditions (21% O_2_), and demonstrated that both compartments are affected by the oxygen tension. In particular, an increase in monounsaturated fatty acids (MUFA) and omega-3 eicosapentaenoic acid (EPA), with decrease in docosapentaenoic acid (DPA), occurred in the membrane phospholipids, confirming that oxygen promotes enzymatic transformations of the fatty acid pool and triggers membrane remodeling [41]. Previous work in human tumor xenografted mice showed a profound lipid remodeling during tumor and age progression consisting of PUFA diminution and SFA enrichment, whereas lipidome reorganization also correlated with some of the markers of DNA damage [42].

In the frame of our interest for CS syndrome, we present here the study on the fatty acid-based membrane lipidome of normal and defective CSA and CSB-transformed fibroblasts, aiming at depicting a comprehensive vision of the remodeling of this cell compartment in response to three experimental oxygen concentrations. In vivo, mammalian cells reside in an environment of 0.5–10% O_2_ (depending on the tissue location within the body), whilst standard in vitro cell culture is carried out under the atmospheric oxygen tension of 21% (what we termed hyperoxia) [43]. In particular, we adjusted the atmosphere to have 21%, 5% and 1% of O_2_ and we refer to them as hyperoxia, physioxia and hypoxia, respectively [44,45].

## 2. Materials and Methods

### 2.1. Materials

Sapienic acid (6c-16:1), 8c-18:1 and sebaleic acid (5c,8c-18:2) methyl esters were purchased from Lipidox (Lidingö, Sweden); cis and trans FAME were purchased from Merck (Darmstadt, Germany) and used without further purification; chloroform, methanol, isopropanol, diethyl ether and n-hexane were purchased from Baker (Phillipsburg, NJ, USA) (HPLC grade) and used without further purification. Silica gel analytical thin-layer chromatography (TLC) was performed on Merck silica gel 60 plates, 0.25 mm thickness, and spots were detected by spraying the plate with cerium ammonium sulfate/ammonium molybdate reagent.

### 2.2. Cell Cultures

CSA and CSB SV40-transformed cell lines were established and cultured as previously described [46]. More precisely, an isogenic cell line that expresses the wild-type (wt) CSA protein tagged with the Flag and HA epitopes (CS3BE-wtCSA) was used [33]. The defective counterpart is CS3BE. For CSB cell lines, we used CS1AN-wtCSB (normal CSB cells) and CS1AN (defective CSB cells) [39]. Defective cell lines carry the empty vector. Cell culture studies were grown under standard atmospheric oxygen tension, 21% O_2_ (hyperoxia), 5% O_2_ (normoxia) and 1% O_2_ (hypoxia).

### 2.3. Phospholipid Extraction and Fatty Acid Analysis

The cells were treated to separate them and wash out the culture medium. The counted (1 × 10^6^ of cells) cells were re-suspended in 100 µL 10 mM phosphate buffer in an Eppendorf vial. To the Eppendorf vial, 0.5 mL of distilled water was added, and centrifugation at 14000 rpm for 15 min at 4 °C was applied to obtain the membrane pellet. Lipid extract from the membrane pellet was obtained using 2:1 chloroform/methanol as the organic phase (4 × 4 mL) following the Folch method [47]. The organic layers were dried on anhydrous Na_2_SO_4_, evaporated to dryness, were weighed (0.9–1.1 mg), and then examined by thin-layer chromatography (TLC) in two different eluent conditions: n-hexane/diethyl ether 3/7 (*v*/*v*) to evidence cholesterol presence, and chloroform/methanol/water 8/2/0.2 (*v*/*v*/*v*) to evidence phospholipid classes. TLC visualization was obtained by spraying the plate with 20% solution of phosphomolybdic acid in ethanol and heating the plate. As previously reported in detail [48], we investigated the presence of cholesterol and phospholipids (including phosphatidyl serine, phosphatidyl ethanolamine, phosphatidyl choline and phosphatidyl inositol) as well as plasmalogens and sphingomyelins as the major components of membrane lipids (>90%). We did not investigate further for phosphatidic acid, and glycolipids that can account for <10% of the remaining lipid quantities in human fibroblasts. Treatment with 0.5 M solution of KOH in MeOH (0.5 mL) for 10 min allowed the conversion of all of the fatty acid residues present as esters of the glycerol moieties of phospholipids and plasmalogens (but not sphingolipids) into fatty acid methyl esters (FAME). FAME are separable by GC which is the gold standard for the separation of saturated and unsaturated fatty acids, including positional and geometrical (trans) isomers, which are the objective of this work. The FAME were extracted with n-hexane (3 × 2 mL), dried on anhydrous Na_2_SO_4_, evaporated to dryness and analyzed by GC in comparison with standard references. The GC (Agilent 6850, Milan) was used in splitless mode, equipped with a 60 m × 0.25 mm × 0.25 µm (50%-cyanopropyl)-methylpolysiloxane column (DB23, Agilent, Santa Clara, CA, USA), and a flame ionization detector with the following oven program: temperature started from 165 °C, held for 3 min, followed by an increase of 1 °C/min up to 195 °C, held for 40 min, followed by a second increase of 10 °C/min up to 240 °C, and held for 10min. A constant pressure mode (29 psi) was chosen with helium as the carrier gas. Methyl esters were identified by comparison with the retention times of authentic samples or trans fatty acid references, obtained as described elsewhere [49,50].

### 2.4. Statistical Analysis

All measurements were performed in triplicate and the data were expressed as mean ± standard deviation (SD). The statistical significance (*p*-values) of the results was calculated by an unpaired two-tailed Student’s t-test using GraphPad Prism™ software version 6.01 for Windows (GraphPad Software Inc., La Jolla, CA, USA). A multiple comparison test was applied to compare the differences among the distinct pair of groups.

## 3. Results

Defective CSA and CSB cells with their normal counterparts were incubated under different oxygen tensions: 21% O_2_ (hyperoxia), 5% O_2_ (physioxia) and 1% O_2_ (hypoxia). After cultivation of the cells, membrane fatty acid composition was determined following known methodologies for the membrane pellet formation, phospholipid isolation and transesterification to obtain fatty acid methyl esters (FAME), analyzed using gas chromatography (GC). We also checked the chemical composition of the pellet that was composed of phospholipids and cholesterol [50]. The methods used in this work for GC analysis have been described elsewhere [49,50]. Three samples for each group were analyzed.

### 3.1. FAME Levels in Normal and Defective CSA Cells

Table 1 shows the composition of 21 membrane fatty acid residues (corresponding to >98% of the peaks present in the analysis), which are reported as relative quantitative percentages (% rel. quant.) of the main peak areas obtained from the GC analyses, calibrated and recognized with appropriate references, as already described. Table 1 also shows the sum of the fatty acids belonging to each fatty acid family (SFA, MUFA, PUFA ω6 and PUFA ω3) present in the membrane phospholipids, together with trans fatty acids (TFA). The components of the ω10 FA family are 6c-16:1 (sapienic acid), 8c-18:1 and 5c,8c-18:2 (sebaleic acid) (cf. Figure 1). The nomenclature used in this work (i.e., 9c-18:1) indicates: (1) the position of the double bond and its geometrical configuration (c or t, cis or trans); (2) the carbon chain length and the number of double bonds (C18:1). Such nomenclature allows for easy following of the biosynthesis shown in Figure 1.

**Figure 2:**
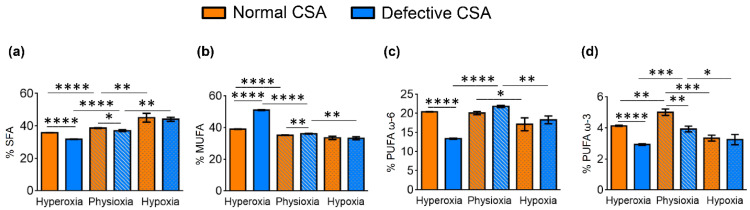
Comparison of the percentages of the fatty acid families (**a**) SFA, (**b**) MUFA, (**c**) PUFA ω-6 and (**d**) PUFA ω-3 in normal (orange bars) and defective (blue bars) CSA cell membrane phospholipids under the three oxygen conditions. The values are given as mean ± SD (*n* = 3). Asterisks indicate the significance of comparisons between normal/defective cells of the same oxygen condition or between physioxia/hyperoxia or physioxia/hypoxia of the same cell line: (*) *p* < 0.05, (**) *p* < 0.01, (***) *p* < 0.001, (****) *p* < 0.0001. For specific values, see Table 1 and Tables S1–S3.

The data in Table 1 can be examined both as the effects of different oxygen concentrations in the same cell line taking the physioxia condition as the comparison for both hyperoxic and hypoxic status, and as the effects of the same oxygen conditions in normal and defective CSA cell lines. Such comparisons and significances (*p*-values) are reported in Tables S1–S3, respectively. In Figure 2, the values of FA families (SFA, MUFA, PUFA ω6 and PUFA ω3) are graphically represented comparing normal (orange) and defective (blue) cell lines, at the three different oxygen tensions, and expressing values as percentage differences together with their statistical significance.

The most relevant changes occurring in normal CSA cells under hyperoxia vs. physioxia are (cf. Table 1 and Table S1):

(i) The significant decrease in SFA (16:0 and 18:0) and significant increase in MUFA (6c-16:1, 9c-16:1, 9c-18:1 and 11c-18:1) production;
(ii) PUFA ω6 increase due to the accelerated transformation of the precursor 18:2 (LA) into 20:3 (DGLA) and 20:4 (ARA) (cf. Figure 1);
(iii) For PUFA ω3, hyperoxic conditions were deleterious for the three components (EPA, DPA, DHA) compared with physioxic conditions;
(iv) It is worth noting that in the normal CSA cells, free radical stress, expressed by the formation of TFA, reached the highest level under hyperoxic conditions. We recall that TFA formation is connected with the radical-catalyzed cis–trans isomerization of the fatty acid double bonds, mainly due to the formation of endogenous sulfur-centered radicals [51,52];
(v) The ω10 FA family, which expresses the Δ6 desaturase activity directly on palmitic acid (cf. Figure 1, left side), increased under hyperoxic conditions, especially with the levels of sapienic (6c-16:1), 8c-18:1 and sebaleic (5c,8c-18:2) acids.

On the other hand, hypoxic conditions on normal CSA determine the following changes compared to physioxia:

(i) A significant increase in SFA (16:0 and 14:0);
(ii) A decrease in ω7 MUFA vaccenic acid (11c-18:1), but an increase in ω10 MUFA (8c-18:1);
(iii) A decrease in PUFA ω6 transformation, in particular to 20:4 (ARA), with accumulation of the precursor linoleic acid, which is not transformed (cf. Figure 1);
(iv) A decrease in PUFA ω3, which was particularly significant for DHA.

The most relevant changes that occurred in the defective CSA cells in terms of hyperoxia vs. physioxia are (cf. Table 1 and Table S2):

(i) The observed changes were similar to the normal cell line under hyperoxia in the SFA–MUFA conversion, with an even more relevant production of 9c-16:1, 9c-18:1, 11c-18:1 and of the ω10 MUFA (6c-16:1 and 8c-18:1);
(ii) For PUFA ω6, hyperoxic conditions led to the diminution of the levels of LA, DGLA and ARA and total ω6;
(iii) PUFA ω3 responded with the diminution of DPA and DHA, whereas EPA increased;
(iv) TFA reached the highest content.

For the defective CSA cells in terms of hypoxia vs. physioxia, the production of MUFA and PUFA was decreased, whereas SFA increased, as observed for the normal CSA cell lines.

Comparing the defective vs. normal CSA cells under the same oxygen tensions (Table 1 and Table S3 and Figure 2), we observed that:

(i) Hypoxia did not create significant fatty acid differences in the FA families;
(ii) Under physioxia, normal cells compared to defective cells had more MUFA ω9 and less SFA, as well as less PUFA ω3, in particular DPA and DHA;
(iii) Hyperoxia created the most relevant changes between the two cell lines, with defective cells showing a decrease in SFA, strong increase in MUFA, decrease in PUFA (except for the increase in ω6 20:2 and the ω3 EPA) and an increase in total TFA.

**Figure 3:**
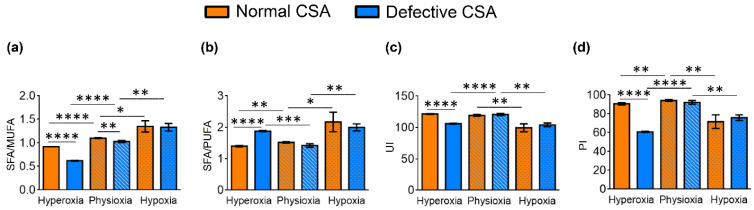
Some membrane homeostasis indexes in normal and defective CSA cells under hypoxic, physioxic and hyperoxic conditions: (**a**) SFA/MUFA ratio; (**b**) SFA/PUFA ratio; (**c**) unsaturation index (UI); and (**d**) peroxidation index (PI). The values are given as mean ± SD (*n* = 3). Asterisks indicate the significance of comparisons between normal/defective cells of the same oxygen condition or between physioxia/hyperoxia or physioxia/hypoxia of the same cell line: (*) *p* < 0.05, (**) *p* < 0.01, (***) *p* < 0.001, (****) *p* < 0.0001. For specific values, see Table 2 and Table S4.

In both the normal and defective CSA cell lines, the hypoxic conditions caused a strong reduction in the unsaturated lipids of both MUFA and PUFA components. In Table 2 and Figure 3, the lipid indexes obtained from the data from Table 1 are shown and can be compared between the oxygen conditions and cell types. The most relevant effects of oxygen tensions in normal cells are: SFA/MUFA and SFA/PUFA ratios decreased in hyperoxia in comparison to physioxia and hypoxia, whereas the ω10 FA increased. In the defective compared to the normal cell line, hyperoxic conditions gave an even lower SFA/MUFA ratio.

Another two important aspects that can be considered are the peroxidation index (PI) and unsaturation index (UI), which can be calculated by Equations (1) and (2), respectively:

UI = (%MUFA × 1) + (%LA × 2) + (%DGLA × 3) + (%ARA × 4) + (%EPA × 5) + (%DHA × 6)(1)

PI = (%MUFA × 0.025) + (%LA × 1) + (%DGLA × 2) + (%ARA × 4) + (%EPA × 6) + (%DHA × 8)(2)

UI and PI indicate the content of unsaturated lipids that have an impact on the membrane properties as MUFA and PUFA double bonds, and on the chemical oxidative reactivity mainly as PUFA double bonds, respectively [53,54]. The PI and UI values for normal and defective CSA cells are shown in Table 2 and are graphically reported in Figure 3c,d, respectively. In normal fibroblasts, the UI and PI indexes were the lowest under hypoxia. The PI value is the lowest under hyperoxia in the CSA-defective cell line.

**Figure 4:**
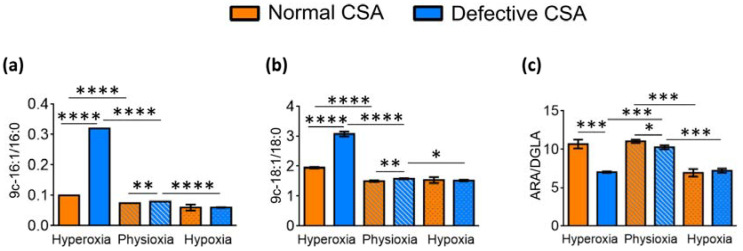
Enzymatic indexes related to desaturase enzymes and defined by the ratio of the associated fatty acids in for normal and defective CSA cell membrane (cf. Table 2): (**a**) Δ9 desaturase index (SCD-16); (**b**) Δ9 desaturase index (SCD-18); and (**c**) Δ5 desaturase index (D5D-16). Asterisks indicate the significance of comparisons between normal/defective cells of the same oxygen condition or between physioxia/hyperoxia or physioxia/hypoxia of the same cell line: (*) *p* < 0.05, (**) *p* < 0.01, (***) *p* < 0.001, (****) *p* < 0.0001. For specific values, see Table 2 and Table S4.

As summarized in Table 2 and Table S4, and clearly depicted in Figure 4a,b, significantly increased Δ9 desaturase (SCD-16 and SCD-18) indexes were reported in the defective compared to the normal cells under hyperoxia (SCD-16; *p* < 0.0001, SCD-18; *p* < 0.0001), with this increase being much smaller (although significant) under physioxia (SCD-16; *p* = 0.0036, SCD-18; *p* = 0.0184). The level of Δ5 desaturase index related to DGLA to ARA transformation (D5D-ARA/DGLA) was instead diminished significantly in hyperoxia when comparing the normal and defective cell lines, as well as in hypoxia compared with physioxia in both the normal and defective cell lines (Figure 4c).

### 3.2. FAME Levels in Normal and Defective CSB Cells

Analogous oxygen conditions were applied to both the normal and defective CSB cell types.

**Figure 5:**
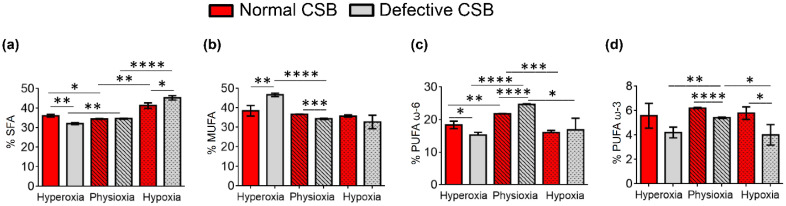
Comparison of the percentages of the fatty acid families (**a**) SFA, (**b**) MUFA, (**c**) PUFA ω-6 and (**d**) PUFA ω-3 in normal (red bars) and defective (gray bars) CSB cell membrane phospholipids under the three oxygen conditions. The values are given as mean ± SD (*n* = 3). Asterisks indicate the significance of comparisons between normal/defective cells of the same oxygen condition or between physioxia/hyperoxia or physioxia/hypoxia of the same cell line: (*) *p* < 0.05, (**) *p* < 0.01, (***) *p* < 0.001, (****) *p* < 0.0001. For specific values, see Table 3 and Tables S5–S7.

It was gratifying to see that the behavior of fatty acid changes and remodeling in this case had some similar trends to the normal and defective CSA cell lines, with a few differences, as underlined below. The results can be examined both as the effects of different oxygen concentrations in the same cell line, and as the effects of the same oxygen conditions in normal and defective CSB cell lines (Table 3 and Figure 5 in the text for the data and Tables S5–S8 in the Supplementary Information for the trends).

The main results for normal CSB cells under hyperoxia vs. physioxia can be summarized as follows:

(i) An increase in SFA and MUFA (9c-16:1 and 9c-18:1) occurred under hyperoxic compared to physioxic conditions, differently from what we observed in the normal CSA cell line (i.e., SFA decrease and MUFA increase);
(ii) PUFA ω6 diminished in hyperoxic conditions;
(iii) PUFA ω3 residues did not show consistent changes;
(iv) As in the previously shown cell lines, TFA were increased under hyperoxia.

For the normal CSB cell lines, hypoxia led to the:

(i) Increase in SFA and increase in MUFA; interestingly, the increase in oleic acid (9c-18:1) was coupled with the decrease in palmitoleic acid (9c-16:1, cf. Figure 1);
(ii) Diminution of PUFA ω6;
(iii) Diminution of TFA.

The main results for the defective CSB cells under hyperoxia vs. physioxia can be summarized as follows:

(i) Hyperoxic conditions gave a boost to the MUFA production in these cell lines, like in the CSA line, and this production was even higher compared to the normal CSB cells, with significantly low levels of SFA;
(ii) PUFA ω6 under hyperoxic conditions was at the lowest levels in the CSB-defective cells, whereas for PUFA ω3, only EPA and DPA were lower compared to the normal CSB cell lines;
(iii) The TFA 9t-18:1 had higher levels under hyperoxia than in the normal cell line.

Under hypoxia, the defective CSB cells showed:

(i) The highest SFA content and the lowest MUFA content, with an increase in two components of the ω10 MUFA, in particular sapienic acid and 8c-18:1;
(ii) Only the PUFA ω3 DHA was significantly low.

**Figure 6:**
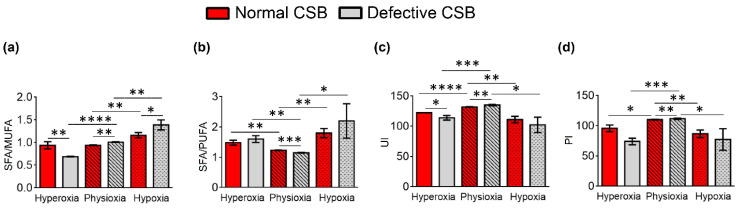
Some membrane homeostasis indexes in normal and defective CSB cells under hypoxic, physioxic and hyperoxic conditions: (**a**) SFA/MUFA ratio; (**b**) SFA/PUFA ratio; (**c**) unsaturation index (UI); and (**d**) peroxidation index (PI). The values are given as mean ± SD (*n* = 3). Asterisks indicate the significance of comparisons between normal/defective cells of the same oxygen condition or between physioxia/hyperoxia or physioxia/hypoxia of the same cell line: (*) *p* < 0.05, (**) *p* < 0.01, (***) *p* < 0.001, (****) *p* < 0.0001. For specific values, see Table 4 and Table S8.

In Table 4 and Figure 6, the lipid indexes obtained from the data of Table 3 are shown and can be compared between the oxygen conditions and cell types. In the normal CSB cells, the SFA/MUFA ratio increased when moving from hyperoxia to hypoxia (Figure 6a), and in the defective cell lines, the trend was similar. In the defective CSB cell line compared to the normal CSB cell line, hyperoxic conditions gave the lowest SFA/MUFA ratio among all conditions and cell types. The SFA/PUFA ratio and the ω10 FA also increased from hyperoxia to hypoxia. Other indicators of the unsaturation behavior are the peroxidation index (PI) and unsaturation index (UI) (cf. Equations (1) and (2)) and are reported in Table 4 [53,54]. Like in the previous CSA cell lines, the UI and PI indexes are significantly lowered by hyperoxia in the CSB-defective cell line. Under hypoxia, compared to hyperoxia, UI and PI decreased in normal cells and the lowest PI value was found in the defective CSB under hyperoxia.

**Figure 7:**
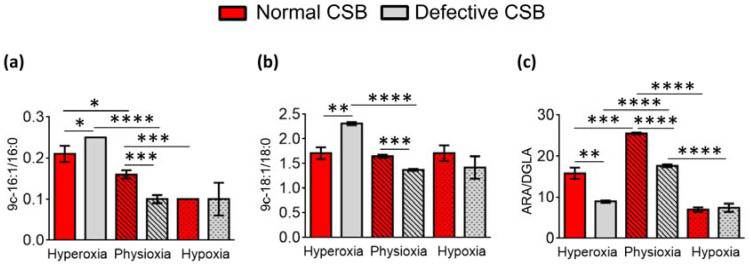
Enzymatic indexes related to desaturase enzymes and defined by the ratio of the associated fatty acids for normal and defective CSB cell membrane (cf. Table 4): (**a**) Δ9 desaturase index (SCD-16); (**b**) Δ9 desaturase index (SCD-18); and (**c**) Δ5 desaturase index (D5D-16). Asterisks indicate the significance of comparisons between normal/defective cells of the same oxygen condition or between physioxia/hyperoxia or physioxia/hypoxia of the same cell line: (*) *p* < 0.05, (**) *p* < 0.01, (***) *p* < 0.001, (****) *p* < 0.0001. For specific values, see Table 4 and Table S8.

As shown in Table 4 and Figure 7, under hyperoxia, the SCD-16 and SCD-18 indexes were significantly raised in the defective compared to the normal CSB cells (SCD-16; *p* = 0.0258; SCD-18; *p* = 0.0011). On the contrary, lower levels of these indexes were observed under physioxia in defective compared to normal CSB cells (SCD-16; *p* = 0.0005; SCD-18, *p* = 0.0005). The SCD-16 index was significantly diminished under hypoxia compared to hyperoxia in normal (*p* = 0.0007) and defective cells (*p* = 0.0029), while SCD-18 was only diminished in defective CSB cells (*p* = 0.0027). A significantly low D5D index (ARA/DGLA) was observed in defective compared to normal CSB cells under hyperoxia (*p* = 0.0010) and physioxia (*p* < 0.0001) and decreased when moving from hyperoxia to physioxia and then to hypoxia in both CSB cell lines.

## 4. Discussion

An extensive study was carried out to examine membrane fatty acid remodeling under three distinct oxygen tensions using two different types of fibroblasts with mutations in the *CSA* and *CSB* genes (CS3BE and CS1AN, respectively) and compared with normal counterparts (CS3BE-wtCSA and CS1AN-wtCSB, respectively). Under oxygen deprivation, the normal fibroblasts of our study showed an increase in SFA residues and a decrease in MUFA in the defective CSA and CSB cell lines. Under hypoxia, the normal CSA cells increased the level of vaccenic acid (11c-18:1) and the normal CSB cells increased the level of oleic acid (9c-18:1). Under hypoxic conditions, the oxygen-dependent fatty acid desaturation pathway could be expected to slow down (see Figure 1), which is one important step in the lipid biosynthesis of eukaryotes [55]. The transformation carried out by delta-9 desaturase (D9D) on SFA, the key step for MUFA biosynthesis, can give information regarding the oxygen availability in the medium. In our experiments, the normal fibroblasts in the CSA and CSB series show delta-9 desaturase activity despite low oxygen tension, whereas the defective cells are not able to respond. On the other hand, under hypoxic conditions, the PUFA ω6 and ω3 levels were diminished in both normal and defective cell lines (except for PUFA ω3 in normal CSB), sometimes reaching lower levels in hypoxia than in hyperoxia (cf., normal CSA cell lines in Table 1 and normal and defective CSB cell lines in Table 3). It is worth noting that PUFA double bonds availability can be connected to oxygen levels in a quite different manner compared to MUFA. Highly unsaturated fatty acids such as ARA, EPA, DPA and DHA can increase under the inhibition of respiration, since under such conditions, both D6D and D5D increase their activity due to more availability of NAD^+^-NADH not used in respiration [56]. However, in our case, this effect was not seen, probably due to the decline of mitochondrial function that characterizes CS syndrome and is derived from the decreased activation of the NAD+/SIRT1/PGC1a axis triggered by the hyperactivation of PARP1 due to chronic oxidative DNA damage [57].

It is also worth recalling at this point that: (i) delta-6 desaturase enzymes are also influenced by the levels of NAD^+^-NADH produced by glycolysis in the cells, and hypoxic conditions are known to slow down aerobic respiration; (ii) in previous work [39], CS cells showed decreased basal rates of oxidative phosphorylation and a concomitant increase in glycolysis. In fact, metabolites involved in anaerobic glycolysis, such as lactate (lac) and alanine (ala), were found to be higher in CS compared to normal fibroblasts. It has therefore been proposed that these metabolic differences may act as possible fingerprints for the pathological status in CS cells [39]. The increased glycolysis in CS cells could be correlated in our experiments to the delta-6 desaturase pathway that produces MUFA ω10 under hypoxia in normal and defective CSA cell lines, as well as in defective CSB cell lines. The MUFA ω10 fatty acid series is emerging for its role in cancer [16,48] and hypoxia is a consistent condition in tumor development [58]. Conversely, the role of hypoxia in the cell regenerative pathway is still debated [59]. The PUFA sebaleic acid could be involved in signaling under hypoxia, as suggested for ARA.

It cannot be excluded that a metabolic reason underlies the decreased PUFA levels due to an increased release of these fatty acids from membranes as precursors of lipid signaling, thus increasing the turnover of PUFA residues for eicosanoid synthesis, as occurs under hypoxia in myocyte cell lines [60]. A second variable influencing the HUFA levels is the occurrence of oxidative or free radical processes, in particular lipid peroxidation, which degrades PUFA-containing 4, 5 and 6 double bonds such as ARA, EPA and DHA, respectively. However, PUFA diminution under hypoxic conditions cannot be explained, since oxidative conditions should not be operative. An old report on the incubation of human aorta under hypoxic conditions showed an increased eicosatrienoic acid synthesis, but not ARA, as detected by ^14^C labeling, leading to the hypothesis of a blockage of the delta-5 desaturation step [61]. In our case, hypoxic conditions seem to have caused the blockage of both D6D and D5D in our fibroblast cell lines, with a stronger effect observed in the defective cell lines than in normal ones. The PUFA ω3 series was also shown to be diminished under hypoxia, again indicating the strong oxygen dependence of the desaturase working on these PUFA series, which overcomes what could be expected on the basis of decreased respiration conditions, as reported previously [56]. The SFA increase in membranes under hypoxic conditions can exert an effect on membrane properties such as fluidity and permeability [62], and less penetration of oxygen can slow down the oxygen-dependent fatty acid desaturation pathway (see Figure 1) that is one important step in the lipid biosynthesis of eukaryotes, whereas prokaryotes can work anaerobically [55]. Conclusively, under hypoxic conditions, the occurrence of oxidative or free radical processes, in particular lipid peroxidation, which degrades PUFA containing 4, 5 and 6 double bonds such as ARA, EPA and DHA, respectively, cannot be evoked. Therefore, the most feasible reason for MUFA and PUFA diminution is enzymatic impairment.

Considering the results under hyperoxic conditions, an increase in MUFA can occur for a boost of biosynthesis from the SCD-1 activation. Both normal and defective CSA and CSB cell lines showed that hyperoxia brings a decisive shift to MUFA. At the same time, the fate of the SFA family, a precursor of MUFA, is not always in diminution. Only normal CSA fibroblasts show diminution of SFA, which is not significant in defective CSA, whereas in normal and defective CSB, the SFA series increased. The normal CSA also exhibited different behavior regarding PUFA levels. In the normal CSA cell line, PUFA ω6 showed an increased production of ARA, whereas the PUFA ω3 series was slightly affected by diminution. In normal CSB, both PUFA ω6 and ω3 (EPA and DPA) diminished, indicating a contribution in this the scenario from an increased oxidative environment in this cell line compared to the CSA cells. It is worth emphasizing that, in the case of increased oxygen levels, not only are desaturase enzymatic activities expected to increase, but also the PUFA reactivity by lipid peroxidation, with the opposite result of decreasing PUFA levels. In this respect, defective cell lines are expected to provide more information on the absence of the CSA or CSB proteins, which is known to lead to higher oxidative damage both in models and in patients [39]. For the defective CSA and CSB cell lines, is evident from the comparisons of the data reported in Table 1 and Table 3 and the corresponding *p*-values reported in Tables S1, S3, S5 and S7 that hyperoxic conditions gave the most striking differences in MUFA biosynthesis, which was strongly increased (51% MUFA in defective CSA vs. 46% MUFA in defective CSB), paired with the loss of the PUFA ω6 and ω3 series. These results suggest taking into account the role of membrane remodeling based on the availability of fatty acids from the cellular lipid pools, which in hyperoxia favors MUFA vs. PUFA residues. This molecular change produces a change of related membrane-based signaling to be deepened in further studies, especially when supplementations of fat and other co-factors in Cockayne syndrome models are carried out [63,64]. The higher loss of PUFA in defective cell lines with a high sensitivity to hyperoxic conditions is an important result in view of the known fast aging process that occurs in Cockayne syndrome, and can be coupled with other reported lipid metabolism changes in murine models [65].

Indeed, the differences reported here between the two types of cell lines and their defective counterparts under different oxygen tensions can inspire further experiments influencing their nutritional fat and cofactor status, in order to follow up the fate of membrane-based signaling. It is interesting to note that data have been reported on the amelioration of Cockayne syndrome’s premature aging in murine models fed a high-fat diet and NAD^+^, which was especially effective in the case of CSB^m/m^ mice [63]. Certainly, the loss of PUFA residues in neurodegenerative diseases suggests the need to proceed with adequate PUFA supplementations, with such lipids being essential for correct cell and tissue functioning [66].

Various therapeutic strategies have been proposed to correct the molecular defects of CS, including, among others: the inhibition of accumulated mitochondrial serine proteases to restore mitochondrial DNA polymerase-γ levels [67]; the reduction of endoplasmatic reticulum stress by chemical chaperones to rescue RNA polymerase I activity and protein synthesis [36]; anti-TNFα therapeutic approaches to prevent apoptosis [68,69] and delay the accelerated aging phenotype [70]; a ketogenic diet to stimulate mitochondrial biogenesis [63]; HDAC inhibitors or overexpression of Parkin to improve autophagic and mitophagic function [38,71]; and a DRP1 inhibitor to mitigate mitochondrial dysfunction and apoptotic rate [37]. Many of these strategies work by controlling the oxidative stress, therefore it is plausible to think that an antioxidant diet could be beneficial for CS patients. In previous work, we reported that treatment with an antioxidant reduces the ROS levels and changes the metabolic profile of CS cells [39]. The results indicate that a multi-targeted strategy could be useful in complex diseases, combining antioxidant protection and appropriate lipid replacement therapy, to intervene synergically on the damages and changes observed in cells compared to controls.

In this work, the fatty acid metabolism of the ω10 FA series is a new aspect that is considered in hyperoxia and hypoxia. In hyperoxia, the total ω10 levels were increased in normal CSA, leading to the highest ω10 FA content in normal CSA (3.9%, Table 2) compared to the other two conditions, and with sebaleic acid reaching almost 1% (rel. quant) in the membrane lipidome (cf., Table 2 and Table S2 in the Supporting Information). This result indicates that in hyperoxic conditions, the CSA cell line activated delta-6 desaturase activity on palmitic to sebaleic acid, as shown in Figure 1, and this is a new aspect of the fatty acid metabolism in this cell line. It is interesting to note that the normal CSB cell line did not respond with such a pathway under hyperoxic conditions, but the defective CSB cell line only responded with the MUFA ω10 FA and not with the PUFA sebaleic acid. We recently published the connection between the ω10 FA series and the signaling cascade starting from the epithelial growth factor receptor (EGFR) [48], and it can be hypothesized that the production of signaling from this fatty acid series can accompany the regeneration of tissues. In the present results, the ω10 FA level makes a difference between normal CSA and CSB; however, a full explanation of this behavior cannot be provided at this point.

Finally, another interesting point is the formation of trans fatty acids (TFA) by the thiyl radical-catalyzed process that occurs under oxygen tensions. [51,72]. In our experiments, we evaluated the trans isomers of MUFA (sapienic and oleic acids), together with the mono-trans isomers of PUFA ω6 linoleic and arachidonic acids (see Table 1 and Table 3) [73]. It is also known that the presence of trans arachidonic acid isomers can inhibit superoxide production by NADPH-oxidase enzymes [74], therefore there is also a connection with the oxidative reactivity under different oxygen tensions. The behavior is relevant in the case of defective cell lines, with the highest levels (1.1%) under hyperoxia and the lowest levels (0.38%) under hypoxia in the defective CSA cell line. Whether this behavior is connected with the thiol levels or metabolism and with differences between normal and defective conditions remains to be determined. These results confirm that the extent of radical-based reactivity is higher in defective cells lines and, among them, the CSA cell lines exhibit a higher reactivity of the fatty acid double bonds toward thiyl radicals.

## 5. Conclusions

We used cell models of a genetic disease, Cockayne syndrome, known to induce high oxidative stress and DNA damage due to the impairment of the DNA repair machinery. After having demonstrated the involvement of DNA damage in these cell models, we examined the response of the phospholipid components involved in the formation of another important cellular compartment, the cell membrane, and observed the results of fatty acid remodeling under hyperoxic (21% O_2_) and hypoxic (1% O_2_) conditions compared to physioxia (5% O_2_) for the first time. The analysis of fatty acid residues in membrane phospholipids evidenced a complex scenario that includes the role of enzymatic functioning, such as desaturase enzymes that work with oxygen, with some unexpected results regarding the reactivity of polyunsaturated components, which are not straightforwardly degraded under high oxygen conditions, as could be expected. Hypoxia evidenced the role of membrane PUFA, especially ω6, which is suggested to be implicated in the signaling mechanism. The CSA and CSB deficiency influenced the behavior at different oxygen tensions, evidencing that CSB cells are the most sensitive cells for the oxidative damage of lipid components, whereas CSA cells were found to have an increased formation of TFA, probably due to the thiol metabolism with thiyl radical formation. Our results suggest a deepening of the membrane behavior when the effects of fat and antioxidant dietary components are evaluated in genetic diseases involving changes of redox status.

## Figures and Tables

**Table 1:** Relative percentage (% rel.) of fatty acid methyl esters (FAME) from normal and defective CSA cells under hyperoxic, physioxic and hypoxic conditions ^1^.

FAME	Normal CSA Cells			Defective CSA Cells		
	Hyperoxia	Physioxia	Hypoxia	Hyperoxia	Physioxia	Hypoxia
14:0	1.40 ± 0.02	0.96 ± 0.29	1.42 ± 0.14	1.45 ± 0.01	0.81 ± 0.25	1.08 ± 0.15
16:0	22.06 ± 0.05	22.72 ± 0.33	28.71 ± 2.41	21.09 ± 0.06	21.63 ± 0.58	27.80 ± 1.37
18:0	12.31 ± 0.09	15.03 ± 0.40	14.93 ± 0.92	9.24 ± 0.19	14.64 ± 0.16	15.24 ± 0.33
SFA	35.77 ± 0.05	38.71 ± 0.18	45.06 ± 2.67	31.79 ± 0.14	37.08 ± 0.63	44.12 ± 1.31
6c-16:1	2.48 ± 0.02	1.91 ± 0.12	2.23 ± 0.14	1.80 ± 0.01	1.55 ± 0.13	1.90 ± 0.28
9c-16:1	2.75 ± 0.05	1.71 ± 0.10	1.69 ± 0.21	6.74 ± 0.02	1.76 ± 0.11	1.62 ± 0.05
8c-18:1	0.47 ± 0.02	0.36 ± 0.09	0.69 ± 0.12	0.68 ± 0.14	0.53 ± 0.15	0.53 ± 0.09
9c-18:1	23.99 ± 0.09	22.51 ± 0.18	22.90 ± 0.77	28.42 ± 0.16	23.10 ± 0.23	23.11 ± 0.84
11c-18:1	8.73 ± 0.10	8.13 ± 0.09	5.70 ± 0.62	12.55 ± 0.19	8.49 ± 0.17	5.79 ± 0.36
11c-20:1	0.31 ± 0.02	0.61 ± 0.05	0.30 ± 0.15	0.66 ± 0.00	0.69 ± 0.07	0.35 ± 0.09
MUFA	39.03 ± 0.15	35.24 ± 0.11	33.51 ± 1.08	51.08 ± 0.24	36.12 ± 0.26	33.29 ± 0.97
18:2 (LA)	4.94 ± 0.05	5.67 ± 0.04	5.99 ± 0.14	1.57 ± 0.01	5.50 ± 0.06	5.67 ± 0.13
20:2	0.49 ± 0.00	0.70 ± 0.04	0.41 ± 0.20	0.88 ± 0.02	0.73 ± 0.02	0.39 ± 0.13
20:3 (DGLA)	1.23 ± 0.05	1.12 ± 0.03	1.35 ± 0.13	1.25 ± 0.02	1.35 ± 0.05	1.49 ± 0.10
20:4 (ARA)	13.08 ± 0.16	12.40 ± 0.09	9.41 ± 1.42	8.81 ± 0.10	13.81 ± 0.18	10.78 ± 0.84
PUFA ω6	20.43 ± 0.09	20.10 ± 0.41	17.16 ± 1.67	13.38 ± 0.15	21.85 ± 0.29	18.33 ± 1.04
20:5 (EPA)	0.26 ± 0.01	0.32 ± 0.16	0.09 ± 0.04	0.47 ± 0.01	0.29 ± 0.06	0.15 ± 0.07
22:5 (DPA)	1.33 ± 0.04	1.66 ± 0.04	1.25 ± 0.30	0.69 ± 0.01	1.50 ± 0.07	1.23 ± 0.19
22:6 (DHA)	2.55 ± 0.02	3.03 ± 0.02	2.01 ± 0.07	1.77 ± 0.03	2.14 ± 0.08	1.87 ± 0.20
PUFA ω3	4.14 ± 0.04	5.01 ± 0.21	3.35 ± 0.19	2.93 ± 0.05	3.93 ± 0.19	3.25 ± 0.33
6t-16:1	0.22 ± 0.01	0.15 ± 0.13	0.16 ± 0.03	0.13 ± 0.01	0.23 ± 0.04	0.18 ± 0.03
9t-18:1	0.07 ± 0.05	0.04 ± 0.07	0.05 ± 0.01	0.11 ± 0.01	0.05 ± 0.07	0.06 ± 0.03
mt 18:2-ω6 ^2^	0.49 ± 0.00	0.16 ± 0.14	0.10 ± 0.02	0.76 ± 0.01	0.23 ± 0.06	0.06 ± 0.02
mt 20:4-ω6 ^2^	0.20 ± 0.07	0.18 ± 0.17	0.16 ± 0.10	0.11 ± 0.03	0.24 ± 0.04	0.08 ± 0.03
TFA	0.98 ± 0.01	0.54 ± 0.47	0.48 ± 0.12	1.11 ± 0.03	0.75 ± 0.09	0.38 ± 0.02
5c,8c-18:2	0.94 ± 0.26	0.60 ± 0.05	0.46 ± 0.02	0.58 ± 0.16	0.74 ± 0.12	0.53 ± 0.07

^1^ The values are obtained from the GC analysis of FAME, derived from cell membrane phospholipids as reported in the experimental part. All measurements were performed in triplicate per group and the values are given as mean ± SD. For graphical presentation of FAME, see Figure S1. ^2^ mt: mono-trans; mt 18-ω6 is the sum of two mono-trans isomers, whereas mt 20:4-ω6 is the sum of four mono-trans isomers.

**Table 2:** Relative percentage (% rel.) of fatty acid families and enzymatic indexes from normal and defective CSA cells under hyperoxic, physioxic and hypoxic conditions.

Entry	Normal CSA Cells			Defective CSA Cells		
	Hyperoxia	Physioxia	Hypoxia	Hyperoxia	Physioxia	Hypoxia
PUFA	25.06 ± 0.12	25.51 ± 0.28	20.97 ± 1.85	17.07 ± 0.20	26.05 ± 0.56	22.11 ± 0.77
SFA/MUFA	0.92 ± 0.001	1.10 ± 0.01	1.35 ± 0.12	0.62 ± 0.01	1.03 ± 0.02	1.33 ± 0.08
SFA/PUFA	1.40 ± 0.02	1.52 ± 0.02	2.17 ± 0.31	1.88 ± 0.02	1.42 ± 0.06	2.00 ± 0.11
PUFA ω6/ω3	4.94 ± 0.0494	4.02 ± 0.19	5.11 ± 0.22	4.56 ± 0.04	5.56 ± 0.21	5.71 ± 0.89
FA ω10 ^1^	3.89 ± 0.30	2.88 ± 0.17	3.38 ± 0.26	3.05 ± 0.27	2.82 ± 0.14	2.95 ± 0.40
9c-16:1/16:0 ^2^	0.12 ± 0.00	0.075 ± 0.00	0.06 ± 0.01	0.32 ± 0.00	0.08 ± 0.00	0.06 ± 0.001
9c-18:1/18:0 ^3^	1.95 ± 0.02	1.50 ± 0.03	1.54 ± 0.10	3.08 ± 0.08	1.58 ± 0.02	1.52 ± 0.03
ARA/DGLA ^4^	10.68 ± 0.59	11.05 ± 0.23	6.95 ± 0.51	7.04 ± 0.12	10.25 ± 0.27	7.22 ± 0.27
PUFA balance ^5^	0.16 ± 0.001	0.20 ± 0.01	0.16 ± 0.01	0.17 ± 0.001	0.15 ± 0.001	0.15 ± 0.02
UI ^6^	121.51 ± 0.46	119.33 ± 1.34	99.67 ± 6.43	106.17 ± 0.55	120.7 ±1.65	104.18 ± 2.89
PI ^7^	90.72 ± 1.14	94.22 ± 0.80	71.71 ± 7.25	60.82 ± 0.71	92.21 ± 1.99	75.97 ± 2.99

^1^ FA ω10 = %6c-16:1 + %8c-18:1 + %5c,8c-18:2. ^2^ Δ9 desaturase index (SCD-16). ^3^ Δ9 desaturase index (SCD-18). ^4^ Δ5 desaturase index (D5D). ^5^ PUFA balance = ((%EPA + %DHA)/PUFA) × 100. ^6^ See Equation (1). ^7^ See Equation (2).

**Table 3:** Relative percentage (% rel.) of fatty acid methyl esters (FAME) from normal and defective CSB cells under hyperoxic, physioxic and hypoxic conditions ^1^.

FAME	Normal CSB Cells			Defective CSB Cells		
	Hyperoxia	Physioxia	Hypoxia	Hyperoxia	Physioxia	Hypoxia
14:0	0.89 ± 0.07	0.83 ± 0.16	0.84 ± 0.20	0.66 ± 0.10	0.55 ± 0.11	1.50 ± 0.43
16:0	20.37 ± 0.46	19.83 ± 0.20	25.48 ± 1.01	17.87 ± 0.33	18.07 ± 0.23	28.50 ± 0.91
18:0	14.87 ± 0.21	13.84 ± 0.31	15.06 ± 1.05	13.59 ± 0.46	16.05 ± 0.16	15.33 ± 0.41
SFA	36.13 ± 0.67	34.50 ± 0.18	41.38 ± 1.40	32.13 ± 0.47	34.67 ± 0.19	45.33 ± 1.13
6c-16:1	1.08 ± 0.11	1.26 ± 0.05	1.49 ± 0.30	1.12 ± 0.03	1.56 ± 0.09	2.15 ± 0.28
9c-16:1	4.19 ± 0.32	3.23 ± 0.16	2.66 ± 0.08	4.47 ± 0.09	1.82 ± 0.09	2.84 ± 1.15
8c-18:1	0.21 ± 0.03	0.66 ± 0.30	0.28 ± 0.03	0.17 ± 0.05	0.34 ± 0.02	0.49 ± 0.10
9c-18:1	25.35 ± 1.65	22.89 ± 0.32	25.68 ± 0.71	31.36 ± 0.76	22.05 ± 0.19	21.66 ± 2.99
11c-18:1	7.58 ± 0.84	8.36 ± 0.01	5.47 ± 0.23	9.28 ± 0.22	8.23 ± 0.10	5.30 ± 0.79
11c-20:1	0.28 ± 0.02	0.34 ± 0.04	0.20 ± 0.09	0.48 ± 0.01	0.42 ± 0.05	0.31 ± 0.14
MUFA	38.49 ± 2.69	36.74 ± 0.13	35.78 ± 0.60	46.71 ± 0.88	34.41 ± 0.29	32.75 ± 3.45
18:2-ω6 (LA)	4.71 ± 0.31	6.10 ± 0.05	5.51 ± 0.44	4.19 ± 0.17	6.52 ± 0.02	5.27 ± 0.56
20:2	0.44 ± 0.00	0.49 ± 0.01	0.30 ± 0.10	0.52 ± 0.02	0.76 ± 0.02	0.35 ± 0.07
20:3 (DGLA)	0.79 ± 0.02	0.56 ± 0.00	1.29 ± 0.13	1.06 ± 0.04	0.91 ± 0.03	1.32 ± 0.28
20:4 (ARA)	12.42 ± 0.85	14.25 ± 0.09	8.94 ± 0.45	9.50 ± 0.59	16.02 ± 0.20	9.92 ± 2.84
PUFA ω6	18.36 ± 1.14	21.78 ± 0.06	16.04 ± 0.64	15.27 ± 0.82	24.65 ± 0.25	16.87 ± 3.59
20:5 (EPA)	0.46 ± 0.04	0.21 ± 0.02	0.25 ± 0.06	0.58 ± 0.05	0.37 ± 0.02	0.17 ± 0.04
22:5 (DPA)	1.79 ± 0.32	2.12 ± 0.04	1.67 ± 0.16	1.19 ± 0.12	1.81 ± 0.02	1.44 ± 0.30
22:6 (DHA)	3.34 ± 0.66	3.89 ± 0.03	3.88 ± 0.52	2.45 ± 0.27	3.23 ± 0.06	2.41 ± 0.58
PUFA ω3	5.59 ± 1.01	6.22 ± 0.05	5.80 ± 0.51	4.22 ± 0.44	5.42 ± 0.06	4.02 ± 0.84
6t-16:1	0.09 ± 0.05	0.17 ± 0.01	0.12 ± 0.03	0.07 ± 0.06	0.18 ± 0.01	0.13 ± 0.01
9t-18:1	0.08 ± 0.04	0.10 ± 0.01	0.08 ± 0.02	0.20 ± 0.01	0.10 ± 0.01	0.10 ± 0.03
mt 18:2-ω6	0.34 ± 0.01	0.16 ± 0.01	0.17 ± 0.05	0.36 ± 0.02	0.20 ± 0.01	0.12 ± 0.09
mt 20:4-ω6	0.20 ± 0.01	0.23 ± 0.02	0.13 ± 0.03	0.18 ± 0.02	0.24 ± 0.03	0.11 ± 0.01
TFA	0.70 ± 0.09	0.65 ± 0.05	0.51 ± 0.06	0.81 ± 0.05	0.71 ± 0.05	0.45 ± 0.11
5c,8c-18:2	0.53 ± 0.11	0.50 ± 0.05	0.40 ± 0.07	0.69 ± 0.10	0.58 ± 0.10	0.51 ± 0.06

^1^ The values are obtained from the GC analysis of FAME, derived from cell membrane phospholipids as reported in the experimental part. All measurements were performed in triplicate per group and the values are given as mean ± SD. For a graphical presentation of FAME, see Figure S2.

**Table 4:** Relative percentage (% rel.) of fatty acid families and enzymatic indexes from normal and defective CSB cells under hyperoxic, physioxic and hypoxic conditions.

	Normal CSB Cells			Defective CSB Cells		
	Hyperoxia	Physioxia	Hypoxia	Hyperoxia	Physioxia	Hypoxia
PUFA	24.48 ± 2.05	28.12 ± 0.16	22.23 ± 1.13	20.18 ± 1.16	30.21 ± 0.27	21.39 ± 4.43
SFA/MUFA	0.94 ± 0.08	0.94 ± 0.01	1.16 ± 0.06	0.69 ± 0.01	1.01 ± 0.01	1.39 ± 0.1
SFA/PUFA	1.48 ± 0.08	1.23 ± 0.01	1.8 ± 0.15	1.60 ± 0.11	1.15 ± 0.01	2.20 ± 0.57
PUFA ω6/ω3	3.33 ± 0.37	3.50 ± 0.02	2.77 ± 0.14	3.63 ± 0.18	4.55 ± 0.03	4.20 ± 0.36
FA ω10 ^1^	1.82 ± 0.03	2.42 ± 0.39	2.16 ± 0.36	1.98 ± 0.12	2.48 ± 0.14	3.15 ± 0.14
9c-16:1/16:0 ^2^	0.21 ± 0.02	0.16 ± 0.01	0.10 ± 0.00	0.25 ± 0.00	0.10 ± 0.01	0.10 ± 0.04
9c-18:1/18:0 ^3^	1.71 ± 0.12	1.65 ± 0.03	1.71 ± 0.16	2.31 ± 0.03	1.37 ± 0.02	1.42 ± 0.23
ARA/DGLA ^4^	15.83 ± 1.36	25.52 ± 0.21	6.98 ± 0.58	8.96 ± 0.26	17.64 ± 0.33	7.44 ± 0.99
PUFA balance ^5^	0.23 ± 0.10	0.22 ± 0.001	0.26 ± 0.01	0.21 ± 0.01	0.18 ± 0.001	0.19 ± 0.01
UI ^6^	122.33 ± 0.02	131.96 ± 0.44	110.94 ± 5.40	113.88 ± 3.85	135 ± 1.01	102.25 ± 12.71
PI ^7^	95.84 ± 5.47	110.14 ± 0.30	86.72 ± 6.12	74.00 ± 5.43	111.71 ± 1.16	77.39 ± 17.79

^1^ FA ω10 = %6c-16:1 + %8c-18:1 + %5c,8c-18:2. ^2^ Δ9 desaturase index (SCD-16). ^3^ Δ9 desaturase index (SCD-18). ^4^ Δ5 desaturase index (D5D). ^5^ PUFA balance = ((%EPA + %DHA)/PUFA) × 100. ^6^ See Equation (1). ^7^ See Equation (2).

## Data Availability

The data presented in this study are available in this article and supplementary material.

## Supplementary Materials

The following are available online at https://www.mdpi.com/article/10.3390/cells11081286/s1, Table S1: Statistical analysis of FAME in normal CSA cells under various oxygen conditions, Table S2: Statistical analysis of FAME in defective CSA cells under various oxygen conditions, Table S3: Statistical analysis of FAME in normal vs. defective CSA cells, Table S4: Statistical analysis of lipid indexes in normal and defective CSA cells under various oxygen conditions, Table S5: Statistical analysis of FAME in normal CSB cells under various oxygen conditions, Table S6: Statistical analysis of FAME in defective CSB cells under various oxygen conditions, Table S7: Statistical analysis of FAME in normal vs. defective CSB cells, Table S8: Statistical analysis of lipid indexes in normal and defective CSB cells under various oxygen conditions, Figure S1: Graphical presentation of FAME in normal and defective CSA cells under various oxygen conditions, Figure S2: Graphical presentation of FAME in normal and defective CSB cells under various oxygen conditions.

## Abbreviations

ARA, arachidonic acid; CS, Cockayne syndrome; DHA, docosahexaenoic acid; DGLA, dihomo-gamma-linolenic acid; DPA, docosapentaenoic acid; ELO, elongase; EPA, eicosapentaenoic acid; FA, fatty acid; FAME, fatty acid methyl esters; GC, gas chromatography; HPLC, high-pressure liquid chromatography; LA, linoleic acid; LPCAT, lysophospholipid acyltransferase; MUFA, monounsaturated fatty acids; NER, nucleotide excision repair; PI, peroxidation index; PLA2, phospholipase A2; PUFA, polyunsaturated fatty acids; ROS, reactive oxygen species; SFA, saturated fatty acids; TFA, trans fatty acids; TLC, thin-layer chromatography; UFA, unsaturated fatty acids; UI, unsaturation index.
