# Supplementary material for: Effects of Oxygen Tension for Membrane Lipidome Remodeling of Cockayne Syndrome Cell Models
Source: Cells. 2022 Apr 10;11(8):1286. doi: 10.3390/cells11081286 (PMC9032135; doi:10.3390/cells11081286)
Supplement: Supplementary file 1 [file cells-11-01286-s001.zip › cells-1592556-supplementary.pdf]

SUPPORTING INFORMATION

Effects of Oxygen Tension for Membrane Lipidome Remodeling of Cockayne Syndrome Cell Models

Carla Ferreri [1], Anna Sansone [1], Marios G. Krokidis [2], Annalisa Masi [1,3], Barbara Pascucci [3,4], Mariarosaria D'Errico [4] and Chryssostomos Chatgilialoglu [1,5,*]

[1] Istituto per la Sintesi Organica e la Fotoreattività, Consiglio Nazionale delle Ricerche, Via P. Gobetti 101, 40129 Bologna, Italy

[2] Institute of Nanoscience and Nanotechnology, N.C.S.R. "Demokritos", 15310 Agia Paraskevi Attikis, Athens, Greece

[3] Institute of Crystallography, Consiglio Nazionale delle Ricerche, Monterotondo Stazione, 00015 Rome, Italy

[4] Department of Environment and Health, Istituto Superiore di Sanità, Viale Regina Elena 299, 00161 Rome, Italy

[5] Center for Advanced Technologies, Adam Mickiewicz University, 61-614 Poznań, Poland

* Correspondence: chrys@isof.cnr.it

Table S1. Statistically significant trends (increase↑ or decrease↓) of fatty acids and families in membrane phospholipids of normal CSA cells under indicated oxygen conditions. The analysis of FAME was carried out as reported in Material and Methods. Significance: (*) $p < 0.05$, (**) $p < 0.01$, (***) $p < 0.001$, (****) $p < 0.0001$.

	normal CSA cells	
**FAME**	**Physioxia vs Hyperoxia**	**Physioxia vs Hypoxia**
14:0	NS	NS
16:0	↓* p=0.0267	↑*p=0.0237
18:0	↓*** p=0.0003	NS
SFA	↓**** p < 0.0001	↑** p =0.0147
6c-16:1	↑ ** p =0.0130	↑*p=0.0397
9c-16:1	↑**** p < 0.0001	NS
8c-18:1	NS	↑*p=0.0189
9c-18:1	↑*** p=0.0002	NS
11c-18:1	↑** p=0.1500	↓**p=0.0026
11c-20:1	↓ *** p =0.0006	↓* p=0.0274
MUFA	↓**** p < 0.0001	NS
18:2	↓**** p < 0.0001	↑*p=0.0190
20:2	↓*** p =0.0002	NS
20:3	↑* p=0.0150	↑*p=0.0405
20:4	↑** p=0.0030	↓* p=0.0215
PUFA ω6	NS	↓* p=0.0415
20:5	NS	NS
22:5	↓*** p =0.0005	NS
22:6	↓**** p < 0.0001	↓**** p < 0.0001
PUFA ω3	↓** p=0.0021	↓*** p=0.0005
6t-16:1	NS	NS
9t-18:1	NS	NS
mt 18:2	↑* p=0.015	NS
mt 20:4	NS	NS
TFA	NS	NS
5c,8c-18:2	NS	↓* p =0.0108

Table S2. Statistically significant trends (increase↑ or decrease↓) of fatty acids and families in membrane phospholipids of defective CSA cells under indicated oxygen conditions. The analysis of FAME was carried out as reported in Material and Methods. Significance: (*) p < 0.05, (**) p < 0.01, (***) p < 0.001, (****) p < 0.0001.

	defective CSA cells	
**FAME**	**Physioxia vs Hyperoxia**	**Physioxia vs Hypoxia**
14:0	↑* p =0.0114	NS
16:0	NS	↑*p =0.020
18:0	↓**** p < 0.0001	↑*p =0.0472
SFA	↓**** p < 0.0001	↑**p=0.0011
6c-16:1	↑* p =0.0293	NS
9c-16:1	↑**** p < 0.0001	NS
8c-18:1	NS	NS
9c-18:1	↑**** p < 0.0001	NS
11c-18:1	↑**** p < 0.0001	↓*** p =0.0003
11c-20:1	NS	↓** p =0.0067
MUFA	↑**** p < 0.0001	↓** p =0.0082
18:2	↓**** p < 0.0001	NS
20:2	↑*** p =0.0008	↓* p =0.010
20:3	↓*p =0.0324	NS
20:4	↓**** p < 0.0001	↓** p =0.0036
PUFA ω6	↓**** p < 0.0001	↓** p =0.0048
20:5	↑** p =0.0016	↓* p =0.036
22:5	↓**** p < 0.0001	NS
22:6	↓**p=0.0017	NS
PUFA ω3	↓***p=0.0009	↓* p =0.0365
6t-16:1	↓*p =0.0137	NS
9t-18:1	NS	NS
mt 18:2 t	↑*** p =0.0001	↓** p =0.0096
mt 20:4-ω6	↓*p =0.018	↓** p =0.0052
TFA	↑** p =0.0028	↓** p =0.0023
5c,8c-18:2	NS	NS

Table S3. Statistically significant trends (increase↑ or decrease↓) of fatty acids and families in membrane phospholipids of normal vs. defective CSA cells under indicated oxygen conditions. The analysis of FAME was carried out as reported in Material and Methods. Significance: (*) $p < 0.05$, (**) $p < 0.01$, (***) $p < 0.001$, (****) $p < 0.0001$.

	**normal vs. defective CSA cells**		
**FAME**	**Hyperoxia**	**Physioxia**	**Hypoxia**
14:0	↑* p = 0.0179	NS	↓* p = 0.0445
16:0	↓**** p < 0.0001	↓* p = 0.0474	NS
18:0	↓**** p < 0.0001	NS	NS
SFA	↓**** p < 0.0001	↓* p = 0.0126	NS
6c-16:1	↓**** p < 0.0001	↓* p = 0.0244	NS
9c-16:1	↑**** p < 0.0001	NS	NS
8c-18:1	NS	NS	NS
9c-18:1	↑**** p < 0.0001	↑* p = 0.0249	NS
11c-18:1	↑**** p < 0.0001	↑* p = 0.0316	NS
11c-20:1	↑**** p < 0.0001	NS	NS
MUFA	↑**** p < 0.0001	↑** p = 0.0026	NS
18:2	↓**** p < 0.0001	↓* p = 0.0151	↓* p = 0.0441
20:2	↑**** p < 0.0001	NS	NS
20:3	NS	↑** p = 0.0022	NS
20:4	↓**** p < 0.0001	↑*** p = 0.0003	NS
PUFA ω6	↓**** p < 0.0001	NS	NS
20:5	↑**** p < 0.0001	NS	NS
22:5	↓**** p < 0.0001	↓* p = 0.0264	NS
22:6	↓**** p < 0.0001	↓**** p < 0.0001	NS
PUFA ω3	↓**** p < 0.0001	↓** p = 0.0027	NS
6t-16:1	↓*** pv = 0.0004	NS	NS
9t-18:1	NS	NS	NS
mt 18:2	↑**** p < 0.0001	NS	NS
mt 20:4	NS	NS	NS
TFA	↑*** p = 0.0028	NS	NS
5c,8c-18:2	NS	NS	NS

Table S4. Statistically significant trends (increase↑ or decrease↓) of lipid indexes in normal and defective CSA cells under indicated oxygen conditions. Significance: (*) p < 0.05, (**) p < 0.01, (***) p < 0.001, (****) p < 0.0001.

Entry	normal vs defective CSA		
	**Hyperoxia**	**Physioxia**	**Hypoxia**
PUFA	↓**** p < 0.0001	NS	NS
SFA/MUFA	↓**** p < 0.0001	↓** p = 0.0056	NS
SFA/PUFA	↑**** p < 0.0001	NS	NS
PUFA ω6/ω3	↓** p = 0.0003	NS	NS
PUFA ω10	NS	NS	NS
9c-16:1 /16:0 9c-18:1 /18:0	↑**** p < 0.0001	↑** p = 0.0036	NS
6c-16:1 /16:0	↑**** p < 0.0001	↑** p = 0.0184	NS
ARA /DGLA	↓**** p < 0.0001	↓*** p = 0.0003	NS
5c,8c-18:2/8c-18:1	↓*** p = 0.0005	↓* p = 0.0174	NS
PUFA BALANCE	↓* p = 0.0186	NS	↑* p = 0.0469
UI	↑*** p = 0.0003	↓*** p = 0.0010	NS
PI	↓**** p < 0.0001	NS	NS
	↓**** p < 0.0001	NS	NS

FAME	normal CSA		defective CSA	
	**Physioxia vs Hyperoxia**	**Physioxia vs Hypoxia**	**Physioxia vs Hyperoxia**	**Physioxia vs Hypoxia**
PUFA	NS	↓* p=0.0137	↓**** p < 0.0001	↓** p =0.0020
SFA/MUFA	↓**** p < 0.0001	↑* p =0.0228	↓**** p < 0.0001	↑**p=0.0032
SFA/PUFA	↓** p=0.0018	↑* p =0.0223	↑*** p =0.0002	↑**p=0.0013
PUFA ω6/ω3	↑** p=0.0013	↑** p =0.0029	↓**p=0.0013	NS
PUFA ω10	↑** p=0.0071	↑* p =0.0494	NS	NS
9c-16:1 /16:0	↑**** p < 0.0001	NS	↑**** p < 0.0001	↓**** p < 0.0001
9c-18:1 /18:0	↑**** p < 0.0001	NS	↑**** p < 0.0001	↓* p =0.049
6c-16:1 /16:0	↑**** p < 0.0001	NS	↑**** p < 0.0001	NS
ARA /DGLA	NS	↓*** p=0.0002	↓***p=0.0009	↓*** p =0.0002
5c,8c-18:2/8c-18:1	NS	↓**p =0.0093	↓*p=0.0188	NS
PUFA BALANCE	↓** p=0.0023	↓**p =0.0080	↑**** p < 0.0001	NS
UI	NS	↓**p =0.0066	↓**** p < 0.0001	↓** p =0.0010
PI	↓** p=0.0012	↓**p =0.0059	↓**** p < 0.0001	↓** p =0.0014

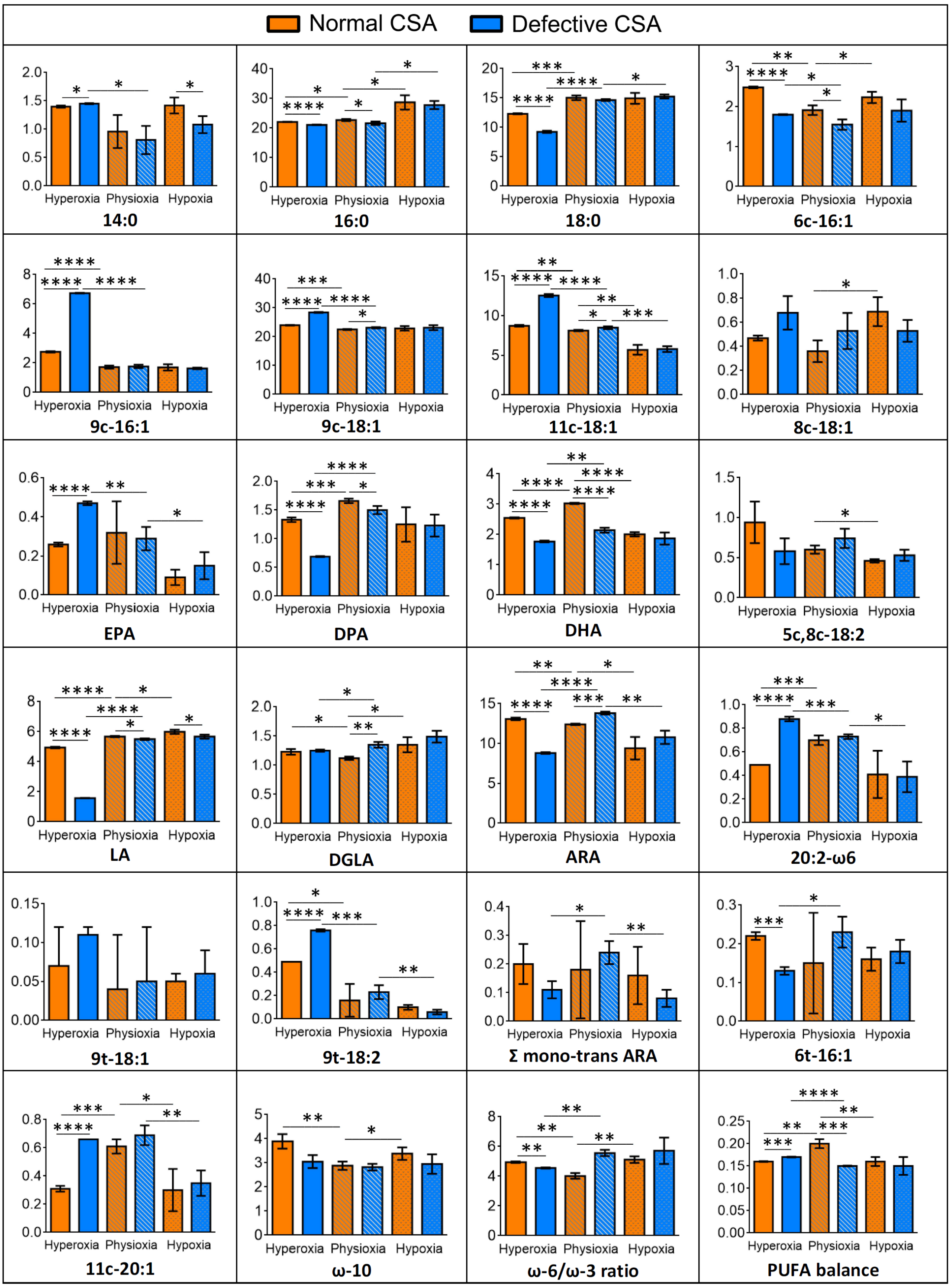

Figure S1. Graphical presentation of FAME (Tables 1 and S1-S3) and some lipid indexes (Tables 2 and S4) for normal and defective CSA cells. The values are given as mean ± SD (n=3). Asterisks indicate the significance of comparisons between normal/defective cells of the same oxygen condition or between physioxia/hyperoxia or physioxia/hypoxia of the same cell line: (*) $p < 0.05$, (**) $p < 0.01$, (***) $p < 0.001$, (****) $p < 0.0001$.

Table S5. Statistically significant trends (increase↑ or decrease↓) of fatty acids and families of normal CSB cells under indicated oxygen conditions. The analysis of FAME was carried out as reported in Material and Methods. Significance: (*) $p < 0.05$, (**) $p < 0.01$, (***) $p < 0.001$, (****) $p < 0.0001$.

	**normal CSB cells**	
**FAME**	**Physioxia vs Hyperoxia**	**Physioxia vs Hypoxia**
14:0	NS	NS
16:0	NS	↑***p=0.0007
18:0	↑** p =0.0089	NS
SFA	↑* pv =0.0150	↑**p=0.0011
6c-16:1	NS	NS
9c-16:1	↑* p =0.0097	↓**p =0.0053
8c-18:1	NS	NS
9c-18:1	NS	↑**p=0.0034
11c-18:1	NS	↓**** p < 0.0001
11c-20:1	NS	NS
MUFA	NS	NS
18:2	↓**p=0.0016	NS
20:2	↓**** p < 0.0001	↓*p =0.0307
20:3	↑**** p < 0.0001	↑***p=0.0006
20:4	↓*p=0.0207	↓**** p < 0.0001
PUFA ω6	↓**p=0.0660	↓*** p =0.0001
20:5	↑*** p =0.0006	NS
22:5	NS	↓** p =0.0091
22:6	NS	NS
PUFA ω3	NS	NS
6t-16:1	NS	NS
9t-18:1	NS	NS
mt 18:2	↑**** p < 0.0001	NS
mt 20:4	NS	↓** p =0.0086
TFA	NS	↓* p =0.0361
5c,8c-18:2	NS	NS

Table S6. Statistically significant trends (increase↑ or decrease↓) of fatty acids and families of defective CSB cells under indicated oxygen conditions. The analysis of FAME was carried out as reported in Material and Methods. Significance: (*) p < 0.05, (**) p < 0.01, (***) p < 0.001, (****) p < 0.0001.

	**defective CSB cells**	
**FAME**	**Physioxia vs Hyperoxia**	**Physioxia vs Hypoxia**
14:0	NS	↑*p =0.0207
16:0	NS	↑**** p < 0.0001
18:0	↓***p=0.0009	↓* p =0.0472
SFA	↓** p =0.0010	↑**** p < 0.0001
6c-16:1	↓**p =0.0013	↑*p =0.0255
9c-16:1	↑**** p < 0.0001	NS
8c-18:1	↓**p =0.0054	NS
9c-18:1	↑**** p < 0.0001	NS
11c-18:1	↑** p =0.0017	↓**p =0.0031
11c-20:1	NS	NS
MUFA	↑ **** p < 0.0001	NS
18:2-ω6	↓**** p < 0.0001	↓*p =0.0181
20:2-ω6	↓*** p =0.0001	↓***p =0.0006
20:3-ω6	↑** p =0.0065	NS
20:4-ω6	↓**** p < 0.0001	↓*p =0.0206
PUFA ω6	↓**** p < 0.0001	↓* p =0.0200
20:5	↑** p =0.0025	↓**p =0.0015
22:5	↓*** p =0.0009	NS
22:6	↓** p =0.0081	NS
PUFA ω3	↓** p =0.0094	↓* p =0.0450
6t-16:1	↓*p =0.0351	NS
9t-18:1	↑*** p =0.0003	NS
mt 18:2	↑*** p =0.0002	NS
mt 20:4	↓* p =0.049	↓**p =0.0021
Total TFA	NS	↓* p =0.024
5c,8c-18:2	NS	NS

Table S7. Statistically significant trends (increase↑ or decrease↓) of fatty acids and families of normal and defective CSB cells under indicated oxygen conditions. The analysis of FAME was carried out as reported in Material and Methods. Significance: (*) $p < 0.05$, (**) $p < 0.01$, (***) $p < 0.001$, (****) $p < 0.0001$.

	**normal and defective CSB cells**		
**FAME**	**Hyperoxia**	**Physioxia**	**Hypoxia**
14:0	↓* p = 0.0130	NS	NS
16:0	↓** p = 0.0016	↓*** p = 0.0003	↑* p = 0.0183
18:0	↓* p = 0.0118	↑ *** p = 0.0003	NS
SFA	↓** p = 0.0011	NS	↑* p = 0.0191
6c-16:1	NS	↑** p = 0.0072	↑* p = 0.0183
9c-16:1	NS	↓*** p = 0.0002	NS
8c-18:1	NS	NS	↑* p = 0.253
9c-18:1	↑** p = 0.0046	↓* p = 0.0174	NS
11c-18:1	↑* p = 0.0275	NS	NS
11c-20:1	↑*** p = 0.0001	NS	NS
MUFA	↑** p = 0.0073	↓*** p = 0.0003	NS
18:2	NS	↑*** p = 0.0002	NS
20:2	↑** p = 0.0023	↑**** p < 0.0001	NS
20:3	↑*** p = 0.0005	↑**** p < 0.0001	NS
20:4	↓** p = 0.0081	↑*** p = 0.0002	NS
PUFA ω6	↓* p = 0.0189	↑**** p < 0.0001	NS
20:5	↑* p = 0.0315	↑*** p = 0.0003	NS
22:5	↓* p = 0.0384	↓*** p = 0.0003	NS
22:6	NS	↓**** p < 0.0001	↓* p = 0.0308
PUFA ω3	NS	↓**** p < 0.0001	↓* p = 0.0349
6t-16:1	NS	NS	NS
9t-18:1	↑ ** p = 0.0073	NS	NS
mt 18:2	NS	↑** p = 0.0080	NS
mt 20:4	NS	NS	NS
TFA	NS	NS	NS
5c,8c-18:2	NS	NS	NS

Table S8. Statistically significant trends (increase↑ or decrease↓) of families and enzymatic indexes in normal and defective CSB cells under indicated oxygen conditions. Significance: (*) p < 0.05, (**) p < 0.01, (***) p < 0.001, (****) p < 0.0001.

Index	normal vs defective CSB		
	**Hyperoxia**	**Physioxia**	**Hypoxia**
PUFA	↓* p = 0.0341	↑*** p = 0.0003	NS
SFA/MUFA	↓** p = 0.0058	↑** p = 0.0003	↑* p = 0.0360
SFA/PUFA	NS	↓*** p = 0.0006	NS
PUFA ω6/ω3	NS	NS	↑** p = 0.0030
PUFA ω10	NS	NS	↑* p = 0.1130
9c-16:1 /16:0	↑* p = 0.0258	↓*** p = 0.0005	NS
9c-18:1 /18:0	↑** p = 0.0011	↓*** p = 0.0005	NS
6c-16:1 /16:0	↑*** p = 0.0003	↑**** p < 0.0001	NS
ARA /DGLA	↓** p = 0.0010	↓**** p < 0.0001	NS
5c,8c-18:2/8c-18:1	NS	NS	NS
PUFA BALANCE	NS	↓**** p < 0.0001	↓** p = 0.0010
UI	↓* p = 0.0191	↑** p = 0.0065	NS
PI	↓** p = 0.0080	NS	NS

FAME	normal CSB		defective CSB	
	**Physioxia vs Hyperoxia**	**Physioxia vs Hypoxia**	**Physioxia vs Hyperoxia**	**Physioxia vs Hypoxia**
PUFA	↓*p=0.0304	↓*** p =0.0009	↓*** p =0.0010	↓* p =0.0262
SFA/MUFA	NS	↑**p=0.0033	↓**** p < 0.0001	↑** p =0.0040
SFA/PUFA	↑**p =0.0058	↑**p=0.0028	↑** p =0.0021	↑* p =0.032
PUFA ω6/ω3	NS	↓ *** p =0.0009	↓*** p =0.0009	NS
PUFA ω10	NS	NS	↓** p =0.0093	↑**p=0.0042
9c-16:1 /16:0	↑* p =0.0179	↓*** p =0.0005	↑**** p < 0.0001	NS
9c-18:1 /18:0	NS	NS	↑**** p < 0.0001	NS
6c-16:1 /16:0	↓***p =0.0003	NS	↓**** p < 0.0001	NS
ARA /DGLA	↓***p=0.0003	↓**** p < 0.0001	↓**** p < 0.0001	↓**** p < 0.0001
5c,8c-18:2/8c-18:1	↑* p =0.0374	NS	↑** p =0.0078	NS
PUFA BALANCE	NS	↑**p=0.0023	↑** p =0.0067	NS
UI	↓**** p < 0.0001	↓** p = 0.0026	↓*** p =0.0006	↓* p =0.0113
PI	↓*p=0.016	↓* p = 0.0027	↓*** p =0.0002	↓* p =0.0173

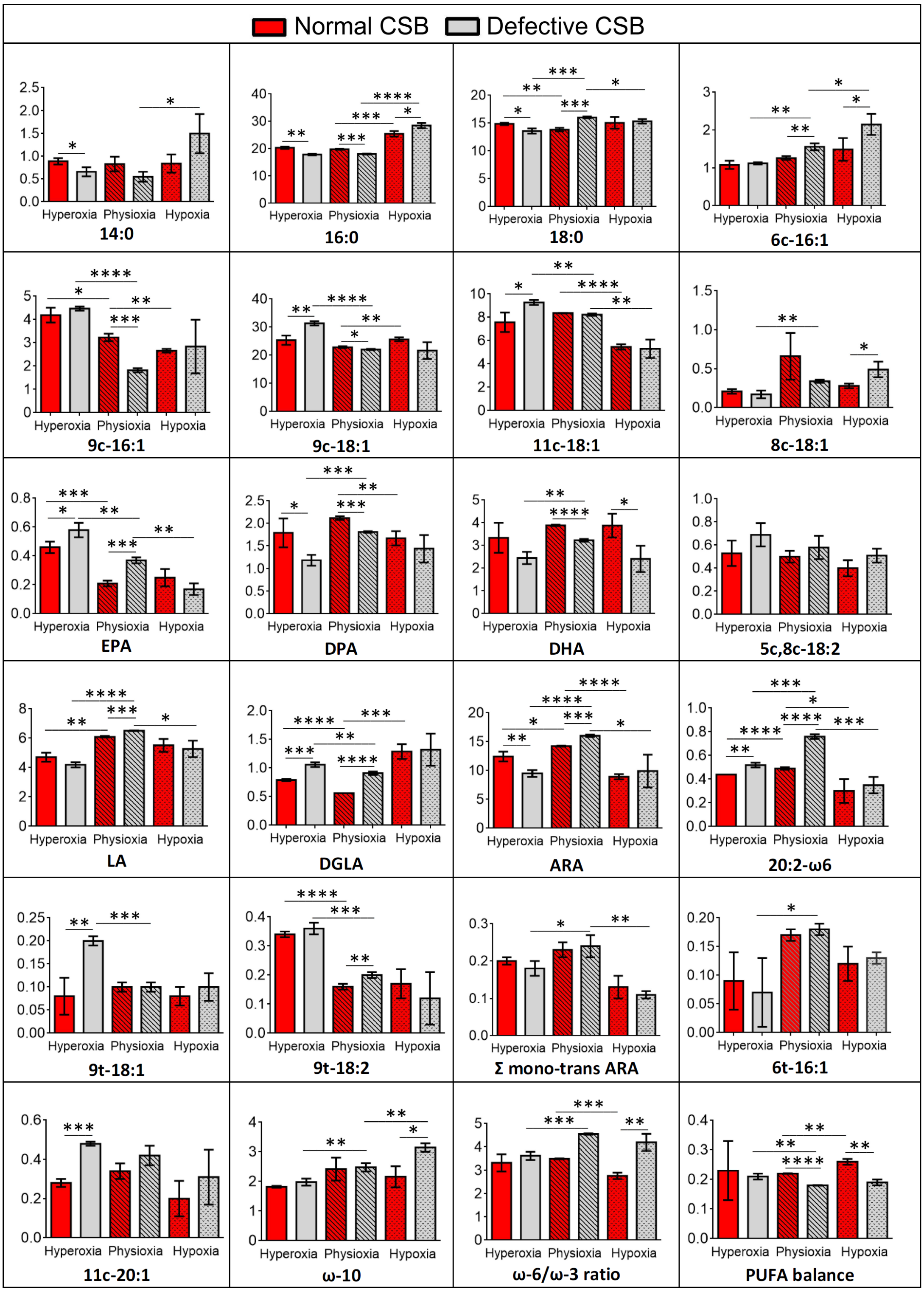

Figure S2. Graphical presentation of FAME (Tables 3 and TS5-S7) and some lipid indexes (Tables 4 and S8) for normal and defective CSB cells. The values are given as mean ± SD (n=3). Asterisks indicate the significance of comparisons between normal/defective cells of the same oxygen condition or between physioxia/hyperoxia or physioxia/ hypoxia of the same cell line: (*) $p < 0.05$, (**) $p < 0.01$, (***) $p < 0.001$, (****) $p < 0.0001$.
